# Peroral endoscopic myotomy: a Danish single center 10-year follow-up study

**DOI:** 10.1007/s00464-025-11832-z

**Published:** 2025-06-16

**Authors:** Martin H. Pedersen, Niels Christian Bjerregaard, Frederik Hvid-Jensen, Daniel W. Kjaer

**Affiliations:** Department of Surgery, Aarhus University Hospital, Palle Juul-Jensens Boulevard 99, Aarhus, Denmark

**Keywords:** Peroral endoscopic myotomy, 10-year follow-up, Gastro-esophageal reflux disease, Satisfaction, Heller’s myotomy, Case-series

## Abstract

**Introduction:**

Achalasia is a rare esophageal motility disorder that in many cases can be treated effectively with Peroral Endoscopic Myotomy (POEM). However, long-term outcomes regarding clinical efficacy, patient satisfaction, and the prevalence of post-POEM gastro-esophageal reflux disease (GERD) remains elusive and thus require further investigation.

**Methods:**

This retrospective cohort study followed 63 patients treated for achalasia with POEM. Clinical success (Eckardt-score ≤ 3, and no subsequent treatments), GERD prevalence (GerdQ-score ≥ 8), and patient satisfaction were assessed via medical record reviews and telephone interviews. Statistical analyses identified risk factors for treatment failure, lower levels of satisfaction, and GERD.

**Results:**

At a median follow-up of 10 years, clinical success was 74%. The average Eckardt-score improved from 7,6 pre-POEM to 2,16 (*p* < 0.0001). The majority of treatment failures occurred within three months post-POEM, with no new failures after 5 years. GERD symptoms were reported by 33% of patients. Patient satisfaction was high with 91% reporting to be satisfied or very satisfied. Treatment-naïve patients had higher success rates (85%) compared to those with prior Heller’s myotomy (40%, *p* < 0.0001). No other risk factors for clinical failure were found. RePOEM showed superior outcomes for salvage treatment compared to balloon dilation, botulinum toxin injections, and Heller’s myotomy.

**Conclusion:**

POEM has a good and lasting efficacy at a median follow-up of 10 years. Clinical failure was not observed beyond 5 years post-POEM. The majority of patients were satisfied with POEM at follow-up. Symptomatic GERD was a highly experienced side effect at follow-up, however, not associated with lower levels of satisfaction.

**Supplementary Information:**

The online version contains supplementary material available at 10.1007/s00464-025-11832-z.

Achalasia is a disease affecting the lower esophageal sphincter (LES) and the distal part of the esophagus. Patients with achalasia are unable to relax their LES and additionally have a loss of peristalsis in the distal part of the esophagus [1]. Patients affected will often develop symptoms of dysphagia for both solids and liquids, as well as regurgitation of undigested food or saliva. Furthermore, patients can develop additional symptoms, including upper-abdominal pain and resort to vomiting to clear the esophagus. As a result of this, many patients will have a significant weight loss [1]. In Western populations, the incidence of achalasia has been found to be around 1,6 cases per 100.000 individuals with a prevalence of ten cases per 100.000 individuals, making it a rare disease. Men and women are equally affected, and symptom onset is seen at all ages, however, less often in children [2]. Achalasia is classified into three subtypes: type I, II, and III—all characterized by a loss of peristalsis. For type I, swallowing results in no significant change in esophageal pressurization. For type II, pan-esophageal pressurization is seen in > 20% of swallows. Lastly, for type III distal spastic contractions are seen in > 20% of swallows [3].

Different treatment options for achalasia exist, all with a common focus on breaking the muscle constriction of the LES. Surgically, a Heller’s myotomy (HM) paired with an anti-reflux procedure is a commonly performed treatment [4]. Pneumatic balloon dilation (PBD) and botulinum toxin injections are also commonly used treatment options [5]. A relatively new treatment option is the Peroral Endoscopic Myotomy (POEM), first introduced in 2008 [6]. POEM is a minimally invasive endoscopic procedure, which has proven to be very safe, with a small percentage of patients facing adverse events [7]. Furthermore, the endoscopic approach allows the operator to make the myotomy longer on the esophagus side, which especially for type III achalasia has proven to be important [8]. When compared to HM, POEM has a comparable, perhaps slightly better, success rate after 24 months at 92,7% compared to 90% for HM [9]. Based on prior data published, POEM is the recommended procedure of choice for achalasia type III and an equally recommended procedure of choice for type I and II along with HM or graded PBD [8]. Generally, the initial feedback regarding POEM’s efficacy and safety has been favorable in studies with short and medium-long follow-up (> 5 years) [7, 10]. However, one significantly proven side effect following patients treated with POEM is gastroesophageal reflux disease (GERD) [11]. A large meta-analysis study shows that symptomatic GERD can be seen in 19% of patients post-POEM compared to only 8,8% of patients post-HM with simultaneous anti reflux procedures [12].

Given that POEM is a relatively new procedure, the literature assessing the long-term efficacy of POEM and the prevalence of post-POEM GERD is insufficient. Furthermore, the patient’s level of satisfaction at long-term follow-ups is generally unassessed in the current literature. Therefore, this study aims to assess the following: (1) the long-term efficacy of POEM in patients from a cohort with 8.5–12.5 years follow-up in regard to clinical success and symptom development throughout the follow-up period; (2) The prevalence of symptomatic post-POEM GERD; (3) The subjective satisfaction of patients following POEM at late follow up.

## Patients and methods

### Study design and cohort

This study is a single center retrospective cohort study retrospectively registered by the Danish regional government and granted full ethical approval. All reporting done following the PROCESS 2023 guidelines [13]. The cohort includes the first patients treated for achalasia with POEM at Aarhus University Hospital from 2012 to 2016 (*n* = 77). It includes data collected prior to POEM and data collected at 3-, 12-, and 24- months post-POEM and at follow-up at a median 10 years post-POEM. All patients were diagnosed in-house using high-resolution manometry, barium swallow, and gastroesophageal endoscopy. All patients were operated by the same surgeon. In this period, all patients at our facility diagnosed with achalasia and deemed surgically eligible were treated with POEM. Patients were excluded at follow-up, if they had passed away due to other causes post-POEM (*n* = 5), if they had had surgery on the distal part of the esophagus, not including HM or redo-POEM (rePOEM), post-POEM (*n* = 1), or if they were severely disabled (*n* = 1). This exclusion criterion resulted in 70 patients eligible for follow-up. Full follow-up was then completed in 63 patients, leading to a follow-up rate of 90% at a median follow-up time of 10 years.

### Follow-up and outcomes

Follow-up was performed via telephone interviews. The patients were called without warning. If the call was left unanswered, a message explaining the call was sent alongside the option to arrange a new interview. At follow-up, three outcomes were assessed: (1) Clinical success, defined as an Eckardt-score of ≤ 3 [14] and no subsequent treatment; (2) The prevalence of post-POEM GERD symptoms, defined as GerdQ-score ≥ 8; [15] (3) Patient satisfaction, defined as “very satisfied”, “satisfied”, “neutral”, or “not satisfied”. Both the GerdQ-score and the Eckardt-score are highly rated patient reported outcome scores (PROMs) for assessing respectively the prevalence of GERD and the symptom severity in achalasia patients [14, 15].

### Variables of clinical interest

Information about demographics, clinical history, and patient satisfaction was collected via telephone interviews and systematic medical records review. This information included sex, age, BMI, duration of symptoms, achalasia subtype, smoking status, alcohol intake, length of myotomy on the esophagus and the gastric cardia past the GEJ, surgical complications, ASA-Score, operation-time, days admitted, previous and subsequent treatment with PBD, HM, botulinum toxin injections, and rePOEM, GerdQ-score [15], Eckardt-score [14] and use of proton pump inhibitors (PPIs).

### Surgical procedure

POEM is a four-step endoscopic procedure. The first step is to make an incision on the posterior or anterior wall of the esophageal wall 2–3 cm proximal of the starting point of the intended esophageal myotomy. Secondly, the endoscope enters the submucosal space and starts dissecting the submucosal fibers, creating a tunnel stretching through the Gastro Esophageal Junction (GEJ) and continuing into the submucosal layer of the gastric cardia. Afterwards, the myotomy is performed through the submucosal tunnel, leaving only the mucosa as a barrier between the esophagus lumen and the abdomen/mediastinum. Finally, the incision in the esophageal wall is closed [16].

### Statistical analyses

Normal distribution of the continuous data was assessed using descriptive statistics assessing the values for skewness and kurtosis, both having to fall within the interval of − 1 and 1. Furthermore, Q–Q plots were produced and assessed for normal distribution. Categorical data is reported as numbers and percentages or fractions and percentages. Continuous data is reported as means with standard deviations (± SD) or medians with interquartile range (IQR) or minimum and maximum values (min–max). Comparison of continuous data was done using the non-paired *t*-test with equal variance. Comparison of pre- and post-POEM Eckardt-scores was done using the paired *t*-test. *P*-values of *p* < 0,05 were considered statistically significant. For the outcome “clinical success”, Cox’s proportional hazards model was applied to assess possible risk factors. The model was adjusted for age, sex, and disease duration pre-POEM. For the outcomes “symptomatic post-POEM GERD” and “satisfaction”, unadjusted logistic regression models were applied to assess possible risk factors. All statistical analyses were performed using Stata 18.0.

## Results

### Patient demographics

After follow-up, the cohort consisted of 63 patients with a median follow-up time of 10 years (min–max 8,5–12). The median age at follow-up was 58 years (min–max 23–87). The patients had a mean disease duration of 7,4 years pre-POEM (± SD 7,9). 28 patients were male (44%). In regard to prior treatment, 36 patients were treatment naïve (57%), 25 patients had received prior PBD treatment (40%), 15 patients had received prior HM (24%), and two patients had received prior botulinum toxin injections (3%). 19 patients were diagnosed with type I achalasia (30%), 22 with type II achalasia (35%) and five patients with type III achalasia (8%). 14 patients were undetermined (22%). The mean myotomy length on the esophagus side was 9 cm (± SD 1,6) and 3,7 cm (± SD 0,8) on the gastric side past the GEJ. Twelve patients had non-severe adverse events (pneumoperitoneum, subcutaneous emphysema, or perforated gastric mucosa) (19%), and one patient had severe adverse events with heavy bleeding post-POEM. All baseline characteristics can be seen in Table 1.

**Table 1:**
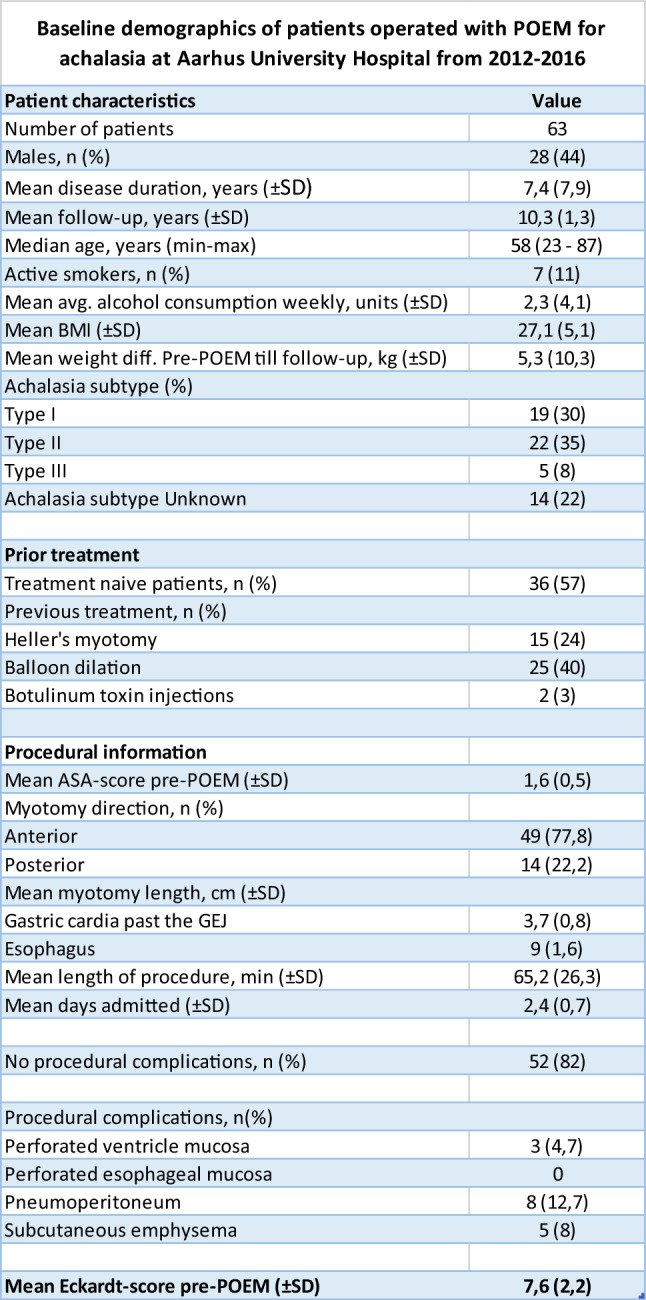
All baseline characteristics for the cohort. Number (*n*), standard deviation (SD)

### Eckardt-score

**Fig. 1:**
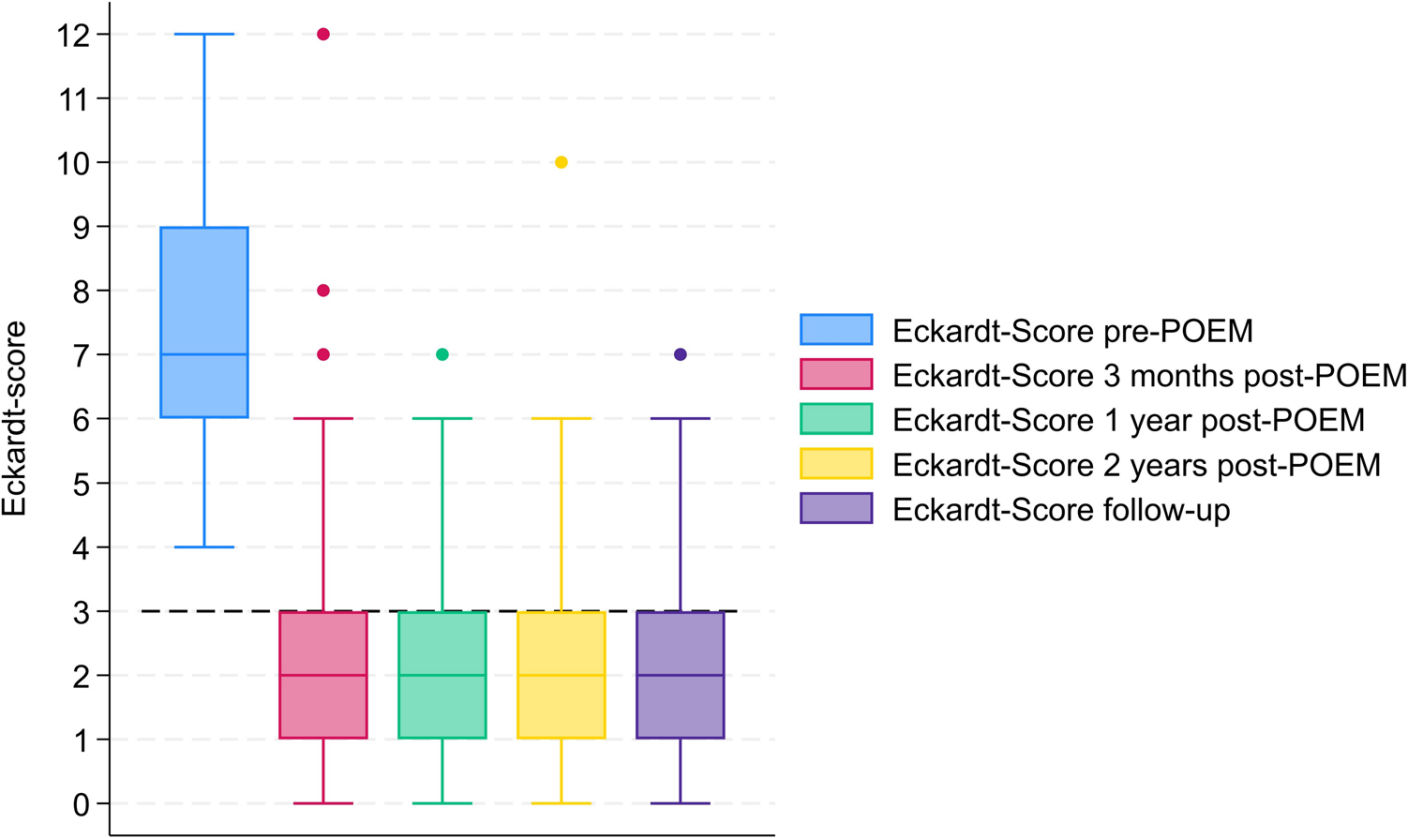
Boxplot showing development in Eckardt-Score at pre-POEM and at all times of follow-up post-POEM. Black stapled line representing Eckardt-score = 3. Boxplot including max–min, IQR, median, and outliers

The mean Eckardt-score (ES) pre-POEM was 7,6 (± SD 2,2). At 3 months post-POEM, the mean ES for all patients had decreased to 2,3 (± SD 2,3). At 1 year, the mean ES was 2,09 (± SD 1,66), at 2 years, it was 2,43 (± SD 1,89), and at follow-up, it was 2,16 (± SD 1,6). The decrease in mean ES from pre-POEM to 3 months post-POEM of 5,3 points was significant (*p* = 0,0001). The change in ES between the postoperative dates was non-significant. All patients but two experienced a decrease in ES from pre-POEM to follow up (97%). Figure 1 illustrates the change in ES from pre-POEM to follow-up for the cohort.

### Clinical success

**Fig. 2:**
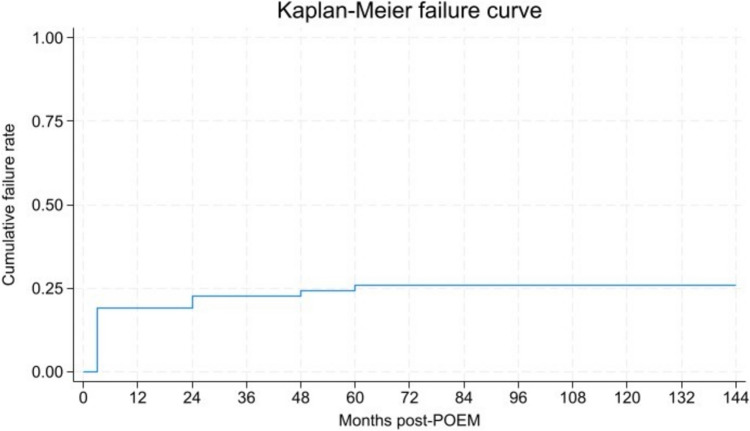
Kaplan Meier failure curve depicting the cumulative failure rate post-POEM with success defined as Eckardt-score ≤ 3 and no subsequent treatment

Initially, at 3 months post-POEM 14 patients (22%) experienced insufficient symptom relief and reported ES > 3. Additionally, seven patients (11%) reported ES > 3 between 1 year post-POEM and follow-up. However, of these patients, five showed spontaneous improvement without the need for additional treatment and were therefore categorized as clinical successes. Conclusively, clinical success was achieved in 47 patients at follow-up (74%). Of the patients with clinical failure at follow up, 12 (19%) were non-responders experiencing clinical failure before three months post-POEM. Four patients (6%) experienced clinical failure between three months post-POEM and follow up. Interestingly, only two patients experienced clinical failure after more than two years (3%), and no patients experienced failure after five years. Among the patients categorized as clinical failure, a significant 3,9-point decrease in ES from pre-POEM to follow-up was still reported (*p* = 0,01). Kaplan–Meier failure curves depicted in Fig. 2.

The success rate was highest among treatment naïve patients at 85% (29/34). For patients previously treated with only PBD or botulinum toxin injections the success rate was 75% (9/12), however, for patients previously treated with HM the success rate was significantly lower at 40% (6/15) when compared to the rest of the cohort (*p* = 0,0001).

Risk factors associated with clinical failure were assessed. Patients previously treated with HM were excluded from the model because their low success-rate disproportionally influenced the overall results. The model was adjusted for age, sex, and disease duration pre-POEM. Neither unadjusted nor adjusted models found any significant risk factors associated with higher risk of clinical failure. All associations can be seen in Table 2.

**Table 2:**
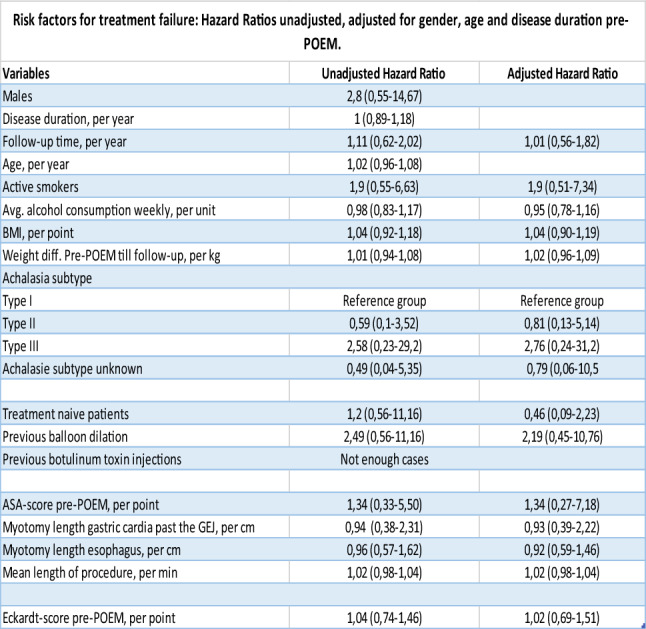
Cox proportional Hazards model testing risk-factors associated with clinical failure, defined as Eckardt-score > 3 or subsequent treatment. Model adjusted for sex, age and disease duration pre-POEM. Hazard Ratio and confidence interval depicted

### Subsequent treatment

At follow up, 16/17 (94%) of the patients with clinical failure had received subsequent treatment. These patients had a mean ES of 3,4 compared to 1,7 in the clinical success group (*p* = 0,0001). However, when compared to the clinical success group, the patients who received rePOEM (*n* = 4) as subsequent treatment also had a higher mean ES of 2 (± SD 2,1), however insignificant (*p* = 0,84). Contrarily, patients who received either HM, PBD, or botulinum toxin injections (*n* = 12) had a significantly higher mean ES of 4 (± SD 1,9) (*p* = 0,0003).

### Reflux symptoms

At follow-up, 21 patients (33%) reported symptomatic post-POEM GERD, and 38 patients (60%) consumed PPIs daily. Univariate logistic regression found no significant risk factors for symptomatic post-POEM GERD. Taking PPIs at follow-up was therefore not associated with prevalent symptomatic post-POEM GERD. However, the only two patients who reported “not satisfied” in regard to the outcome of POEM, mentioned reflux as being the reason for their dissatisfaction. All associations can be seen in Table 3.

**Table 3:**
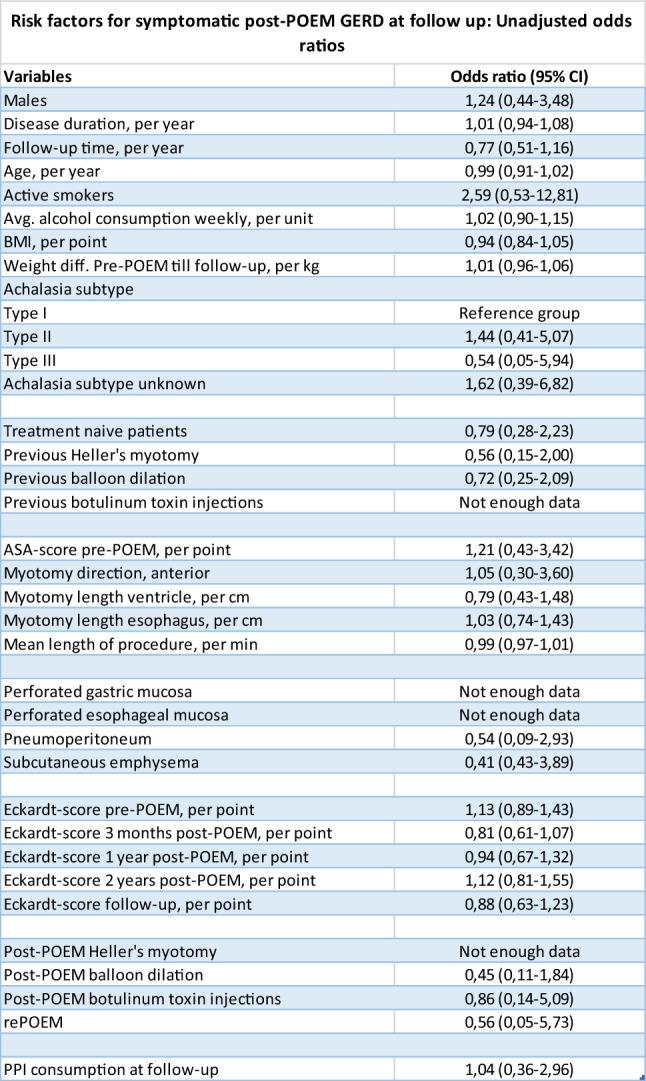
Assessing possible risk factors for symptomatic post-POEM GERD with unadjusted odds ratios with confidence intervals

### Satisfaction

At follow-up, the patients were asked how satisfied they were with their results after POEM. Here 51 patients reported that they were “very satisfied” (81%), six patients reported “satisfied” (10%), four were “neutral” (6%), and two were “not satisfied” (3%). Possible risk-factors for lower levels of satisfaction were investigated. All associations can be seen in Table 4.

**Table 4:**
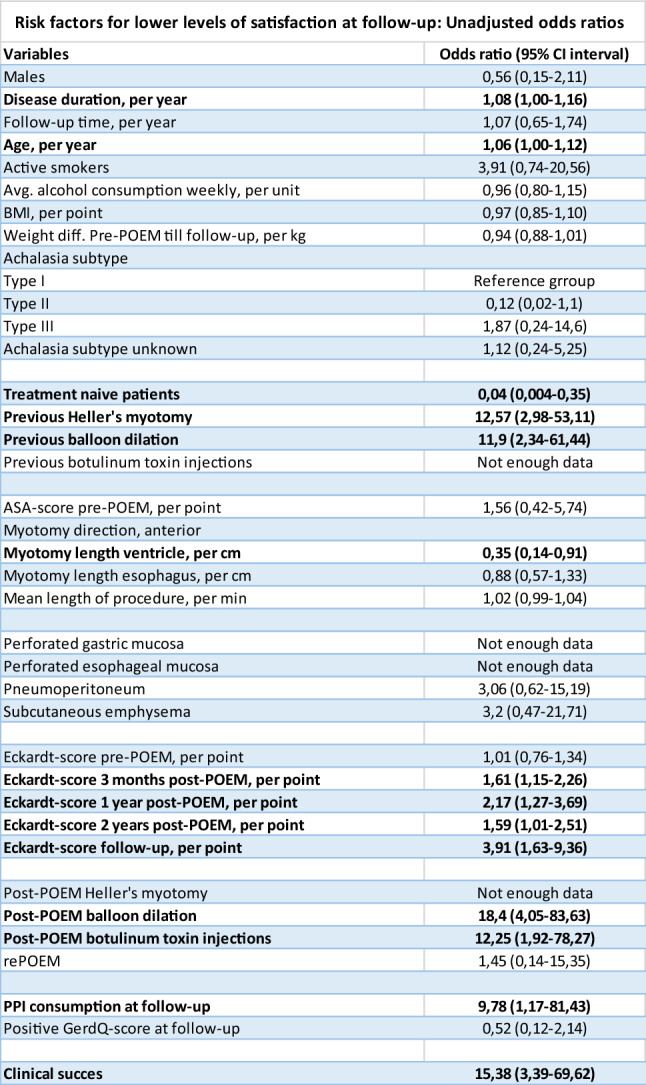
Assessing possible risk factors for lower levels of satisfaction with unadjusted odds ratios with confidence intervals. Significant risk factors written in bold

Both longer disease duration pre-POEM and higher age were significantly associated with lower levels of satisfaction at follow up, (Odds Ratio 1,08, 95% CI 1,00–1,16; OR 1,06, 95% CI 1,00–1,12, respectively) despite neither variables being significantly associated with higher risk of clinical failure or symptomatic post-POEM GERD at follow-up. Unsurprisingly, previous treatment with both HM and PBD were also associated with lower levels of satisfaction. Additionally, shorter myotomy on the gastric side past the GEJ was associated with lower levels of satisfaction. As expected, higher ES at all times of follow up was associated with lower levels of satisfaction. In regard to subsequent treatment, only rePOEM was not associated with lower levels of satisfaction. Surprisingly, PPI consumption at follow-up, but not positive GerdQ-score, was associated with lower levels of satisfaction.

## Discussion

This study aimed to investigate the long-term efficacy of POEM for achalasia patients with a median follow-up of 10 years post-POEM. A significant and lasting decrease in Eckardt-score was observed for the cohort. Only two patients had an unchanged Eckardt-score at follow-up. The decrease in Eckardt-score found in this study is comparable with those reported in other studies with short to medium-long follow-up [17–19] and one with a long follow-up [20]. These findings suggest a good clinical efficacy even at 10 years post-POEM. Furthermore, we found that most failures occurred within the first three months post-POEM, and that no patients experienced failure after five years post-POEM. This underlines the long-term efficacy of POEM in patients with initially good results. Additionally, this is relevant when constructing follow-up guidelines for patients treated with POEM in the future, as the follow-up period for many patients can be shortened.

When assessing our clinical success rate, it is lower than those reported by other studies with medium-long follow up also using ES ≤ 3 and freedom from subsequent treatment as the clinical success criteria. These studies find success rates varying from 79 to 92% [18, 21–23], compared to the 74% observed in this study. However, only between 0 and 10% of their patients had received prior HM compared to 24% in our cohort. For our cohort, we found that the group of patients who had received prior HM had a negative effect on the total success rate, as only 40% (6/15) of those patients met the clinical success criteria at follow-up, compared to 85% (41/48) of the patients who were HM naïve. Generally, POEM is found to be a good salvage operation for previous HM patients, with a comparable efficacy to treatment naïve achalasia patients [21, 24]. However, certain factors may help explain the low clinical success rate observed in our study for this group of patients. Firstly, in our cohort the patients who were previously treated with HM had a significantly longer disease duration pre-POEM than the rest of the patients (see Supplementary Table 1). This suggests that some of these patients may have had more advanced achalasia, which results in higher resistance to treatment with myotomy [25, 26]. Secondly, previous HM is associated with a higher level of technical difficulty [27, 28]. Thirdly, only 15 patients in our cohort had received prior HM, limiting the statistical power of this subgroup analyses. Furthermore, the relatively high proportion of previously treated patients and the associated lower overall success rate, may partly be explained by the patient selection of the cohort. All patients referred to our department with achalasia, whether newly diagnosed or previously treated, were treated with POEM, provided they were deemed surgically eligible. This absence of patient selection could result in a higher number of complex patients with hard-to-treat symptoms, who would thus have a lower likelihood of success. Furthermore, when interpreting the mean ES of 1,7 in our clinical success group, it is worth noting that patients with ES of two or three, still have substantial symptoms and could be classified as marginal clinical success.

In regard to subsequent treatment, we found that both PBD and botulinum toxin injections were significantly associated with lower levels of satisfaction. However, rePOEM was not. The data showed that 75% (3/4) of patients reported to be “very satisfied” after rePOEM and had a mean ES of 2 (± SD 2,1). For patients subsequently treated with PBD or botulinum toxin injections, only 16% (2/12) reported to be “very satisfied” after POEM and reported a mean ES of 4 (± SD 1,9). These findings indicate that rePOEM in our cohort has been a more viable salvage treatment for initially failed POEM than PBD and botulinum toxin injections. In a retrospective multicenter study, Ichkhanian et al. compared salvage treatment for failed POEM and also found rePOEM to be superior to PBD [29]. Other studies have also found rePOEM to be a viable salvage treatment [30, 31]. However, our cohort, including only four patients who received rePOEM and 16 who received subsequent treatment of any kind, is too small to conclude anything significant. Additionally, the argument behind offering one or the other treatment has not been investigated in this study.

When interviewed at follow-up, the majority of patients in our study were satisfied with the procedure (91%). Only 9% (6/63) of patients were not satisfied (4 “neutral” and 2 “not satisfied”). The otherwise high level of satisfaction is a strong indicator of the relevance of POEM and for its enduring efficacy. McKay et al. found a similar level of satisfaction in 100 patients at 5 years follow-up [22].

As anticipated, a higher mean Eckardt-score at all follow-up times post-POEM significantly increased the odds of lower levels of satisfaction. Clinical failure also significantly increased the odds of lower levels of satisfaction at follow-up. Furthermore, higher age, longer disease duration pre-POEM, and shorter myotomy on the gastric side past the GEJ were all found to significantly increase the odds of lower levels of satisfaction. However, longer disease duration pre-POEM is significantly associated with patients who have undergone previous HM (see Supplementary Table 1), which may account for their observed significance as this group of patients had poor results as discussed previously. Interestingly, PPI intake, but not symptomatic post-POEM GERD, was significantly associated with lower levels of satisfaction. This discrepancy may stem from the difficulty in accurately diagnosing symptomatic GERD in achalasia patients, as both conditions share overlapping symptoms such as regurgitation and chest pain, making interpretation challenging [32]. Patients with post-POEM symptoms were likely prescribed PPIs as a precautionary measure, even if their symptoms were not definitively linked to reflux. This could result in an overrepresentation of PPI use among patients with more symptoms, who are also more likely to report lower levels of satisfaction. Additionally, it is relevant to address, that the only two patients who reported to be “not satisfied” with POEM, mentioned reflux as being the reason why. This highlights the importance of post-POEM GERD management even when our analysis shows no association between symptomatic post-POEM GERD and lower levels of satisfaction.

Our study found that 33% (21/63) of patients experienced symptomatic post-POEM GERD, which aligns with results from other studies with shorter follow-up periods [10, 12]. However, as noted earlier, subjectively assessing reflux in achalasia patients is challenging. This is reflected in the wide range of reported prevalence in the literature, varying from 6% to over 40% [12, 33]. No significant risk factors for symptomatic post-POEM GERD were found within this cohort.

### Strengths and limitations

Certain limitations for this study must be acknowledged. Firstly, the cohort included in this study is relatively small at 63 patients all treated at the same center. Secondly, the data collected from patients regarding the outcomes is subjective, possibly leading to information bias. Lastly, these patients represent the first patients treated at Aarhus University Hospital, thus the learning curve must be taken into consideration. In an extensive study, analyzing the learning curve for POEM, Liu et al. found that the learning curve for optimal clinical success was 90–100 cases [34]. Among the study’s strengths, the high follow-up rate of 90% and the 10-year median follow-up time is impressive and highly reduces the risk of selection bias. Future studies could beneficially use more objective parameters for assessing post-POEM GERD such as 24 h-pH monitoring and esophageal endoscopy. However, high follow-up rates could be difficult to maintain increasing the risk of bias.

## Conclusion

Conclusively, this study found that POEM has a good and lasting efficacy at a median follow-up of 10 years. Clinical failure was not observed beyond five years post-POEM. Patients previously treated with a Heller’s myotomy had a significantly lower success rate than the rest of the cohort. No other risk-factors were associated with clinical failure. 91% of patients were satisfied with POEM at follow-up and 33% of patients experienced symptomatic post-POEM GERD at follow-up.

## Supplementary Information

Below is the link to the electronic supplementary material.

Supplementary file1 (DOCX 18 KB)
