# Supplementary material for: Peroral endoscopic myotomy: a Danish single center 10-year follow-up study
Source: Surg Endosc. 2025 Jun 16;39(8):4806–14. doi: 10.1007/s00464-025-11832-z (PMC12287216; doi:10.1007/s00464-025-11832-z)
Supplement: Supplementary file 1 — Supplementary file1 (DOCX 18 KB) [file 464_2025_11832_MOESM1_ESM.docx]

Supplementary table 1: Assessing possible risk factors associated with having received a pre-POEM Heller’s Myotomy with unadjusted odds ratios with confidence intervals. Significant risk factors written in bold.
